# Antihyperlipidaemic effect of triterpenic acid-enriched fraction from *Cyclocarya paliurus* leaves in hyperlipidaemic rats

**DOI:** 10.1080/13880209.2016.1267231

**Published:** 2017-01-31

**Authors:** Zhengfeng Wu, Tianhong Gao, Rongling Zhong, Zi Lin, Cuihua Jiang, Sheng Ouyang, Ming Zhao, Chuntao Che, Jian Zhang, Zhiqi Yin

**Affiliations:** a Department of Natural Medicinal Chemistry & State Key Laboratory of Natural Medicines, China Pharmaceutical University, Nanjing, P.R. China;; b Laboratory of Translational Medicine, Jiangsu Province Academy of Traditional Chinese Medicine, Nanjing, P.R. China;; c Laboratory Animal Research Center, Jiangsu Province Academy of Traditional Chinese Medicine, Nanjing, Jiangsu Province, P.R. China;; d Department of Pharmacy, JiangXi University of Traditional Chinese Medicine, Nanchang, P.R. China;; e Department of Medicinal Chemistry and Pharmacognosy, and WHO Collaborating Center for Tradition Medicine, University of Illinois at Chicago, Chicago, IL, USA

**Keywords:** Apolipoprotein B48, Microsomal triglyceride transfer protein, Tumor necrosis factor-alpha, Mitogen-activated protein kinase

## Abstract

**Context:***Cyclocarya paliurus* (Batal) Iljinskaja (Juglandaceae) is an edible and medicinal plant; the leaves are used in Chinese folkloric medicine to treat dyslipidaemia and diabetes.

**Objective:** This study evaluates the antihyperlipidaemic potential of the triterpenic acid-enriched fraction (TAE) from *C. paliurus* and the underlying mechanism.

**Materials and methods:** The hyperlipidaemic rats were induced by high fat diet for 6 weeks. After oral administration of TAE (200 and 400 mg/kg), the neutral fraction (150 and 300 mg/kg) and statin (4 mg/kg) to the hyperlipidaemic rats for 4 weeks, lipid profile and apolipoprotein (apoB48) level in plasma, and the expression levels of apoB48, microsomal triglyceride transfer protein (MTP), phosphorylation of mitogen-activated protein kinase (MAPK) and tumour necrosis factor α (TNF-α) in intestine were examined. The main constituents in the TAE were identified by HPLC-MS.

**Results:** TAE administration (400 mg/kg) decreased the levels of atherogenic lipids in serum and liver (*p* < 0.05) and increased serum high-density lipoprotein cholesterol by 19.7%. Furthermore, TAE treatment (200 and 400 mg/kg) decreased plasma apoB48 level by 15.3 and 19.5%, downregulated intestinal apoB48 and MTP expression levels (*p* < 0.05), and inhibited TNF-α expression by 36.2 and 56.2% and the phosphorylation level of MAPK by 8.8 and 13.2%, respectively. HPLC analysis revealed the presence of pentacyclic- and tetracyclic-triterpene acids in TAE.

**Conclusion and discussion:** These findings suggested that TAE possessed antihyperlipidaemic activity partially involved in the inhibitory effect on apoB48 overproduction, which may provide evidence about its potential role in ameliorating dyslipidaemia.

## Introduction

Cardiovascular disease is a leading cause of death and it is clinically associated with dyslipidaemia characterized by elevated levels of plasma triglyceride (TG), total cholesterol (TC) and low-density lipoprotein cholesterol (LDL-C), as well as a low concentration of the high-density lipoprotein cholesterol (HDL-C) (Natarajan et al. 2010). Furthermore, the excessive intake of dietary lipids can lead to hyperlipidaemia with an elevated incidence of atherosclerotic disease (Zilversmit 1995).

Apolipoprotein B48 (apoB48) is the constitutive protein of chylomicron, which is involved in dietary lipid absorption (Ros 2000). The overproduction of intestinal apoB48 might contribute to the elevation of atherogenic triglyceride-rich lipoprotein (TRL) particles in the circulation (Cohn et al. 1993) and an elevated risk of coronary artery disease (Masuda et al. 2011; Mori et al. 2013). Microsomal triglyceride transfer protein (MTP) catalyzes the transfer of the lipids to apoB48 in endoplasmic reticulum lumen to assemble chylomicrons. In addition, MTP binds to nascent apoB48 to prevent dislocation from the endoplasmic reticulum and avoid proteosomal degradation (Hussain et al. 2003; Pal et al. 2005).

Epidemiological studies revealed that elevated level of lipoproteins containing apoB could trigger inflammation and initiate atherosclerosis in human subjects and experimental animals (Zilversmit 1995; Glass & Witztum 2001; Mori et al. 2013). Tumour necrosis factor (TNF-α), a major proinflammatory cytokine, also plays a critical role in the lipid metabolism, especially lipid absorption (Chen et al. 2009). TNF-α has been demonstrated to stimulate p38 mitogen-activated protein kinase (MAPK) pathway activation and intestinal production of apoB48-containing lipoprotein (Qin et al. 2010). Moreover, SB203580, the pharmacological p38 inhibitor, could block TNF-α-induced apoB48 oversecretion *in vivo* and *ex vivo* (Qin et al. 2007).

*Cyclocarya paliurus* (Batal) Iljinskaja (Juglandaceae), known as the ‘sweet tea tree’, is an edible and medicinal plant growing on the highlands in Southern China (Shu et al. 1995; Xie & Li 2001). The leaves of the plant are often consumed as a beverage to prevent and treat hyperlipidaemia and diabetes mellitus (Xie & Li 2001). The plant extract has shown therapeutic effects in the treatment of dyslipidaemia, diabetes and hypertension (Ren 1994), as well as the inhibition of lipid peroxidation (Dong et al. 2007).

Polysaccharides, flavonoids and triterpenoids have been isolated from *C. paliurus* (Xie & Xie 2008). However, the active antihyperlipidaemic ingredients remain unknown. Previous work revealed that the water extract could reduce postprandial triglyceride level in hyperlipidaemic mice (Kurihara et al. 2003), and polysaccharides might be the active components (Huang et al. 2011). However, our previous findings demonstrated that the triterpenoids might be the antihyperlipidaemic constituents (Wang et al. 2013a), including cyclocaric acid B, arjunolic acid, corosolic acid, oleanolic acid and ursolic acid which have been isolated from the leaves of *C. paliurus* (Cui & Li 2012). Oleanolic acid and ursolic acid, naturally occurring in a large variety of plants, have been shown to increase the HDL-C level and decrease the atherogenic lipid level (Somova et al. 2003). Corosolic acid is reported to inhibit cholesterol acyltransferase activity (Han et al. 2014). Interestingly, we found that the total extract of *C. paliurus* leaves abundant in triterpernoids decreased the serum level of total apoB48 and the expression of TNF-α in hyperlipidaemic mice (Jiang et al. 2014; Ma et al. 2015).

Therefore, the present study was designed to evaluate the antihyperlipidaemic activity of triterpenic acid-enriched fraction of *C. paliurus* leaves, and probe into its potential mechanism by inhibiting apoB48 secretion in high fat diet (HFD)-induced hyperlipidaemic rats.

## Materials and methods

### Plant material

Fresh leaves of *Cyclocarya paliurus* (Batal) Iljinskaja were collected in October 2010 in the campus of the Nanjing Forestry University, China (GPS coordinates: 118.822414, 32.085054), and was authenticated by Prof. Minjian Qin of the China Pharmaceutical University. Voucher specimen (No. L20100033) was deposited in Department of Natural Medicinal Chemistry of the university.

### Drugs and reagents

Simvastatin tablet from Yichang Changjiang Pharmaceutical Enterprise (Yidu, Hubei, China) was used as a reference antihyperlipidaemic drug. Assay kits for TG, TC, LDL-C, HDL-C, apoB48, superoxide dismutase (SOD), malondialdehyde (MDA), catalase (CAT), and glutathione peroxidase (GSH-Px) were purchased from the Nanjing Jiancheng Bioengineering Institute (Nanjing, Jiangsu, China). Commercial diagnostic kit was obtained from the Nanjing Senbeijia Biological technology Co. (Nanjing, Jiangsu, China) for the determination of serum apoB48. Anti-apoB, anti-MTP, anti-MAPK, anti-phospho-MAPK, horseradish peroxidase-conjugated anti-rabbit and anti-goat antibodies were obtained from the Cell Signaling Technology, Inc. (Beverly, MA). Arjunolic acid, 2α, 3α, 23-trihydroxyursa-12, 20(30)-dien-28-oic acid, cyclocaric acid B, pterocaryoside B, 2α, 3α, 23-trihydroxyurs-12-en-28-oic acid, hederagenin, 3β, 23-dihydroxy-12-ene-28-ursolic acid and oleanolic acid were isolated from our laboratories with >98% purity. All the other chemicals were of analytical grade.

### Preparation and analysis of the plant extracts

The dried and ground leaves (2.5 kg) of *C. paliurus* were extracted with 80% ethanol (3 × 20 L, each for 2 h). The combined extracts were filtered and concentrated under reduced pressure to obtain a semi-solid residue, which was suspended in water, defatted with petroleum ether and partitioned with chloroform to afford a chloroform fraction (171.9 g). The chloroform fraction was dissolved in chloroform and partitioned with 3% NaOH aqueous solution to obtain an organic phase labelled as the neutral fraction (NeE, 56.2 g). The aqueous phase was neutralized with 5% HCl and re-extracted three times with chloroform to provide the fraction enriched in triterpenic acids (TAE, 84.7 g) (Paraschos et al. 2007).

The triterpenic acid-enriched fraction was dissolved in HPLC grade methanol to prepare sample solution (5 mg/mL) for analysis. HPLC analysis was performed on an Agilent 1260 Infinity HPLC system equipped with a UV detector. Chromatographic separation was conducted on an Alltima C18 column (4.6 × 250 mm, 5 μm, from Alltech, Milan, Italy). The solvent system composed of solvent A (acetonitrile) and solvent B (0.01% formic acid in H_2_O) in the following gradient: 0–15 min, 45% A; 15–25 min, 45% A to 52% A; 25–30, 52% A; 30–40 min, 52% to 55% A; 40–50 min, 55% A; 50–80 min, 55% A to 100% A. Operating conditions were as follows: detection wavelength, 205 nm; flow rate, 1.0 mL/min; column temperature, 30 °C; injection volume, 5 μL.

The presence of triterpenoids in TAE was confirmed by a Bruker AmaZon SL LC-MS (Sparta, NJ). The MS conditions were as follows: capillary, 4500 V; end plate offset, −500 V; dry heater temperature, 180 °C; dry gas flow rate, 8.0 L·min^−^^1^; nebulizer pressure, 17.0 psi. The data acquisition was accomplished by Bruker Daltonics for MS^2^/MS^2^ fragmented ions of the components.

### Animals

SD male rats (180–220 g) were purchased from the Experimental Animal Center of Nantong University (Nantong, Jiangsu, China) [Certificate No. SCXK (SU 12014-000)] and acclimatized for 7 days in the animal room. They were kept in controlled ambient temperature (24 ± 2 °C) and humidity (60 ± 10%) under a 12 h light-dark cycle; water was available ad libitum. The study protocol was approved by the Animal Ethics Committee of the Jiangsu Province Hospital of Integration of Chinese and Western Medicine. The animal protocol was approved by the Animal Care Ethics Committee of Southeast University (Dingjiaqiao Campus, Nanjing, Jiangsu, China) [Approval No. 20,140,809].

### Induction of hyperlipidaemia

After the adaptation period, rats were fed with high-fat diet, consisting of 2% cholesterol, 10% lard, 10% egg yolk, 0.5% bile sodium, 77.5% standard diet (w/w) (Pang et al. 2002). After six weeks, the hyperlipidaemic rats showing serum cholesterol level >140 mg/dL were used as described below (Hirunpanich et al. 2006).

### Experimental procedures

**Figure 1.**
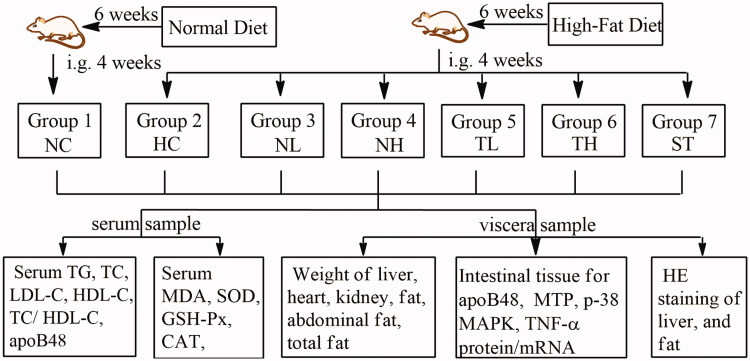
Experimental procedure. High-fat-diet induced hyperlipidaemic rats were treated with *Cyclocarya paliurus* extracts for 4 weeks, following with the determination of lipid metabolism, antioxidant activity, serum apoB48 content and intestinal apoB48, MTP, MAPK and TNF-α expression levels. Groups: normal control (NC), hyperlipidaemic control (HC), hyperlipidaemic rats treated with neutral fraction (NL and NH) at 0.15 and 0.3 g/kg doses and triterpenic acids-enriched fraction (TL and TH) at 0.2 and 0.4 g/kg doses, respectively, and simvastatin tablets (ST).

Animals were divided into 7 groups of 8 rats each as follows (Figure 1):

Group 1: normal control (NC), administered with 0.5% sodium carboxyl methyl cellulose (CMC-Na, 10 mL/kg/day).

Group 2: hyperlipidaemic control (HC), administered with 0.5% CMC-Na (10 mL/kg/day).

Group 3 and 4: hyperlipidaemic rats treated with the triterpenic acids-enriched fraction (TL and TH) at 200 and 400 mg/kg doses, respectively.

Group 5 and 6: hyperlipidaemic rats treated with neutral fraction (NL and NH) at 200 and 400 mg/kg doses, respectively.

Group 7: hyperlipidaemic rats treated with simvastatin (ST, positive control, 4 mg/kg) (Yi et al. 2001; Ma et al. 2015; Wang et al. 2013a).

The test samples were dissolved or suspended in 0.5% CMC-Na. Each group was administered by oral gavage once a day for 4 weeks. Behavioural activity, fur condition, water and food intake of the rats were observed daily. Body weight and food intake were recorded at weekly intervals until the end of the experiment.

### Blood sampling and biochemical analysis

After 4-week treatment, blood samples were drawn after overnight fast from the orbital venous plexus and centrifuged at 3000 rpm for 15 min, and then the serum was stored at −80 °C until analysis.

Serum lipids level indices of TG, TC, HDL-C, LDL-C and apoB48 were measured using commercial assay kits according to the manufacturer’s directions by an automatic biochemical analyzer (Roche Modular DP, Basel, Switzerland). MDA, GSH-Px, CAT and SOD were determined following the standard protocols.

### Analyses of relative organs

At the end of the experiment, overnight-fasted animals were intraperitoneally anesthetized with 10% chloral hydrate (0.3 mL/100 g), and then bloods were collected from the abdominal aorta. The heart, liver, kidney and adipose tissues (including the epididymal, perirenal and abdominal adipose tissues) were removed immediately and weighed after rinsed with physiological saline. They were stored at −80 °C until use.

### Histological evaluation of liver and fat samples by haematoxylin-eosin (HE) staining

The liver and adipose tissues were collected, immediately fixed in 10% neutral formalin for 48 h, dehydrated though the standard procedure, and embedded in low-melting point paraffin wax. Sample sections (5 μm thick) were cut and stained with haematoxylin-eosin solution.

### Western blotting analysis

Intestinal tissue (100 mg) was lysed with cold lysis buffer (0.5 mL, Beyotime, Jiangsu, China) and centrifuged (13,000*g* for 15 min at 4 °C). The supernatants were separated for the measurement of protein concentration using a Bicinchoninic Acid Protein Assay Kit (St. Louis, MO). Proteins (50 μg) were subjected to 5% SDS-PAGE and immunoblotting was performed using Anti-apoB, anti-MTP, anti-MAPK, anti-phospho-MAPK. Then the blots were incubated with secondary horseradish peroxidase-conjugated anti-rabbit antibody. The band intensity was quantized using the IPP 6.0 software (Qin et al. 2009).

### Real-time quantitative reverse-transcriptase polymerase chain reaction (qRT-PCR) analysis

Total RNA samples from small intestines were isolated using Trizol reagent (Invitrogen, Carlsbad, CA). cDNA was synthesized using the Maxima First Strand cDNA Synthesis Kit (Thermo Scientific, Wilmington, DE) with a S1000™Thermal cycler (Bio-Rad, Hercules, CA). Real-time PCR was performed in 25 μL with a Stratagene Mx3000P QPCR System (Agilent Technologies, La Jolla, CA) using the Maxima SYBR Green/ROX qPCR Master Mix (2x) (Thermo Scientific, Wilmington, DE). The program had a UDG pretreatment of 50 °C and an initial incubation of 95 °C for 10 min, followed by 40 cycles of 95 °C for 15 s, 60 °C for 30 s and 72 °C for 30 s. The sense and antisense primers sequence (5eque) for analysis are shown as follows:

apoB: *TGTCTGAAGCCATCTGTAA* and *TCTGAAGAAGCGACTGTT*;

MTP: *AATAATGAGCGGCTATACAAG* and *TTCCTCCACAGTAACACAA*;

TNF-α: *GCCCTACGGGTCATTGAGAG* and *GAGAGACGACAGACGCAGAC*;

β-actin: *GTGGGTATGGGTCAGAAG* and *AAAGTGTGGTGCCAAATC*, respectively.

The gene expression from each sample was analyzed in triplicates. mRNA abundance was calculated according to the 2^-ΔΔCt^ method and normalized to β-actin mRNA (Jiang et al. 2014). Levels of the NC group were arbitrarily assigned a value of 1.

### Statistical analysis

Data are expressed by the means ± standard error. The data were evaluated using the SPSS software package, Version 19.0 (Chicago, IL). Statistical significance was performed using one-way analysis of variance (ANOVA) followed by Turkey *post hoc* test. *p* values <0.05 were considered significant.

## Results

### Phytochemical analysis

**Figure 2.**
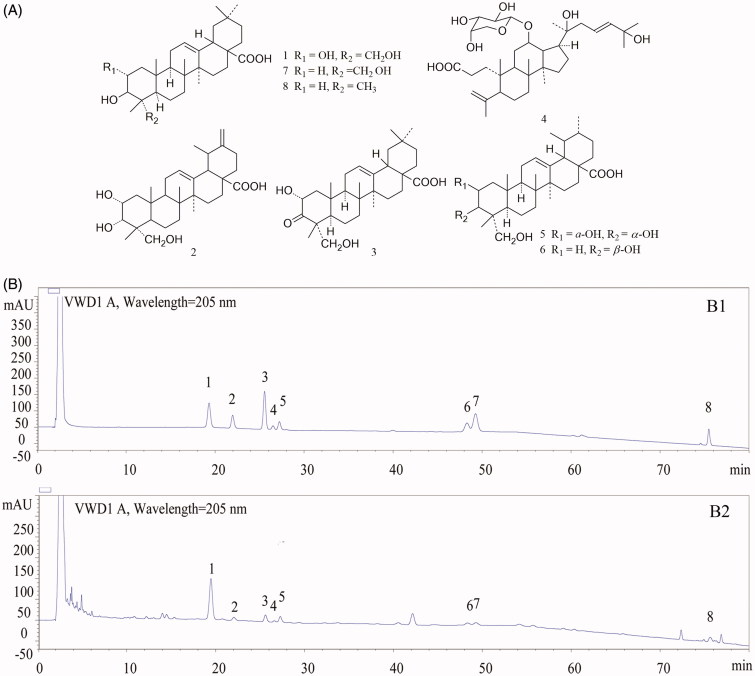
The structures of the compounds (A) and high performance liquid chromatography profiles of standard mixture (B1) as well as the triterpenic acid-enriched fraction (B2) of *Cyclocarya paliurus* using UV detection. External standards: (1) arjunolic acid; (2) 2α, 3α, 23-trihydroxyursa-12, 20(30)-dien-28-oic acid; (3) cyclocaric acid B; (4) pterocaryoside B; (5) 2α, 3α, 23-trihydroxyurs-12-en-28-oic acid; (6) 3β, 23-dihydroxy-12-ene-28-ursolic acid; (7) hederagenin; (8) oleanolic acid.

TAE typical chromatogram was shown in Figure 2(B2) by HPLC analysis. A comparison with corresponding reference standards (Figure 2(B1)) revealed the presence of arjunolic acid, 2α, 3α, 23-trihydroxyursa-12, 20(30)-dien-28-oic acid, cyclocaric acid B, pterocaryoside B, 2α, 3α, 23-trihydroxyurs-12-en-28-oic acid, 3β, 23-dihydroxy-12-ene-28-ursolic acid, hederagenin and oleanolic acid (Figure 2(A)), which was confirmed by LC-MS analysis (Table 1). These results indicated pentacyclic triterpenic acids were the major components of the TAE fraction. The relative content of the individual constituent was presented in Table 1.

**Table 1.** Identification and determination of the compounds in the triterpenic acid-enriched fraction of *Cyclocarya paliurus* by HPLC.

	Retention time (min)				LC-MS		
No.	Reference standard	TAE	Compounds	Molecular weight	[M-H]^−^	[M + Cl]^−^	Contents (mg/g)
1	19.145 ± 0.040	19.211 ± 0.064	Arjunolic acid	488.35	487.35	523.32	211.00 ± 2.53
2	21.755 ± 0.006	21.803 ± 0.035	2α3α,23-Trihydroxyursa-12, 20(30)-dien-28-oic acid	486.33	485.35	521.30	13.18 ± 0.16
3	25.346 ± 0.006	25.387 ± 0.029	Cyclocaric acid B	486.33	485.37	521.33	36.14 ± 0.98
4	26.312 ± 0.012	26.356 ± 0.040	Pterocaryoside B	622.41	621.44	657.38	13.73 ± 0.32
5	27.035 ± 0.012	27.054 ± 0.035	2α,3α,23-Trihydroxyurs-12-en-28-oic acid	488.35	487.35	523.32	30.73 ± 0.47
6	48.997 ± 0.017	47.999 ± 0.006	3β, 23-Dihydroxy-12-ene-28-ursolic acid	472.35	471.34	507.29	29.34 ± 1.21
7	48.912 ± 0.012	48.950 ± 0.034	Hederagenin	472.35	471.35	507.32	21.75 ± 0.31
8	75.431 ± 0.017	75.566 ± 0.029	Oleanolic acid	456.36	455.30	–	36.42 ± 2.04

The retention time and content of the compounds were expressed as a mean of three measurements (mean ± S.E.).

### Body weight, food intake and organ weight

The food consumption of all animals was monitored during the experiment. Rats in the HC group consumed a little less amount of food than that in the NC group presumably because of the unpleasant taste of the high-fat diet (Table 2). However, there was no obvious difference in food intake among the groups fed with HFD.

**Table 2.** Food intake of hyperlipidaemic rats (g, daily average, *n* = 8).

	Before administration (g)			After administration (g)	
Group	2 week	4 week	6 week	2 week	4 week
NC	20.9	19.5	20.0	20.5	20.8
HC	19.6	18.2	19.4	18.9	18.7
NL	19.1	18.9	18.9	18.4	18.1
NH	19.2	18.8	18.7	18.2	18.2
TL	19.3	19.2	19.0	18.3	18.3
TH	18.7	19.9	18.7	17.9	18.1
ST	19.8	17.9	18.6	17.3	18.0

No significant difference was detected in the weight of the heart, liver and kidney in the experimental groups (Table 3). The body weight in the HC rats was significantly less than that in the NC rats, whereas the weight of adipose tissue in the HC group was remarkably higher than that in the NC group (*p* < 0.05). Interestingly, high-dosage of TAE treatment (TH group) led to a significant reduction in body weight, as well as in the weight of adipose tissues including abdominal fat and epididymal fat, compared to the HC group (*p* < 0.05). However, treatment with the neutral fraction has no observable effect on the weight of both body and the adipose tissue compared with the HC group.

**Table 3:** Body weight, weight of organs and adipose tissues (*n* = 8, mean ± S.E.).

Group	Body weight (g)	Organs and adipose tissues weights (g/30 g body weight)					
		Heart	Liver	Kidney	Abdominal fat	Epididymal fat	Perirenal fat
NC	332.2 ± 2.5	0.09 ± 0.003	0.78 ± 0.02	0.20 ± 0.007	0.32 ± 0.01	0.33 ± 0.009	0.10 ± 0.003
HC	303.6 ± 10.3^a^	0.09 ± 0.003	0.80 ± 0.02	0.20 ± 0.005	0.45 ± 0.05^a^	0.44 ± 0.008^a^	0.13 ± 0.002^a^
NL	295.4 ± 11.3^a^	0.10 ± 0.003	0.81 ± 0.03	0.21 ± 0.009	0.41 ± 0.03	0.42 ± 0.004^a^	0.12 ± 0.003
NH	298.6 ± 9.9^a^	0.10 ± 0.003	0.82 ± 0.02	0.21 ± 0.005	0.40 ± 0.03^a^	0.41 ± 0.007	0.12 ± 0.004
TL	291.9 ± 12.2^b^	0.10 ± 0.004	0.79 ± 0.02	0.21 ± 0.009	0.36 ± 0.03	0.40 ± 0.007	0.12 ± 0.003
TH	282.3 ± 5.1^b^^,^^c^	0.09 ± 0.001	0.83 ± 0.02	0.20 ± 0.007	0.33 ± 0.01^c^	0.38 ± 0.005^c^	0.11 ± 0.003
ST	281.6 ± 7.9 ^b^^,^^c^	0.10 ± 0.005	0.74 ± 0.03	0.21 ± 0.006	0.28 ± 0.02^c^	0.39 ± 0.007	0.10 ± 0.002^c^

a *p* < 0.05.

b *p* < 0.01 compared with NC.

c *p* < 0.05 compared with HC.

### Lipid profile in hyperlipidaemic rats

**Figure 3.**
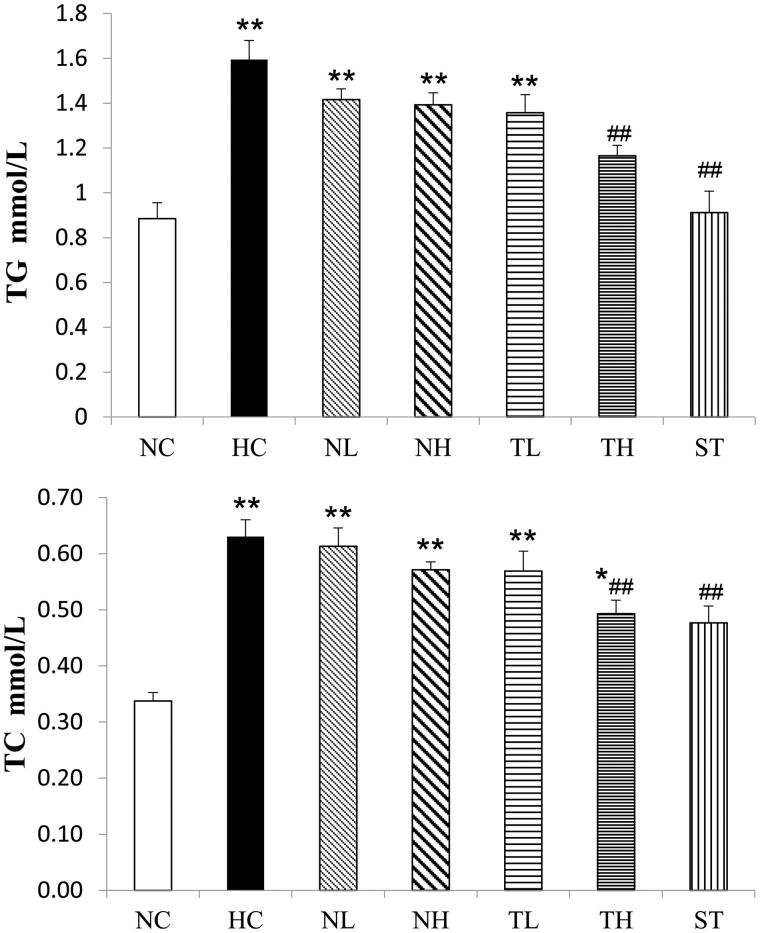
Effects of different extracts on hepatic TG and TC levels in hyperlipidaemic rats (mmol/L, *n* = 8, mean ± S.E.). *p < 0.05, **p < 0.01 compared with NC; #*p* < 0.05, ##*p* < 0.01 compared with HC.

Effects of the plant extracts on serum lipid profiles in the experimental rats are shown in Table 4. It is clear that high-fat diet significantly led to the increased levels of TC, TG and LDL-C, as well as the decreased level of HDL-C compared to the NC group (*p* < 0.01). After TAE treatment, the levels of TC, TG and LDL-C were significantly reduced (*p* < 0.05), while the level of HDL-C was elevated compared to the HC group (TH, 19.7%, *p* < 0.05, respectively). These results suggested TAE administration led to a notable reduction of serum atherogenic index (TC/HDL-C) in treated rats compared with the HC group (TH, *p* < 0.01). However, little change was observed in the NL and NH groups. Additionally, the increased levels of hepatic TC and TG induced by the HFD were notably alleviated in the TH group (*p* < 0.01) (Figure 3), whereas no similar effect was observed in the NL and NH groups.

**Table 4.** Effects of different extracts on lipids and lipoproteins in hyperlipidaemic rats (*n* = 8, mean ± S.E.).

Group	TG(mmol/L)	TC(mmol/L)	LDL-C(mmol/L)	HDL-C(mmol/L)	TC/HDL
NC	0.48 ± 0.04	1.68 ± 0.04	0.29 ± 0.02	2.29 ± 0.05	0.80 ± 0.04
HC	0.72 ± 0.10^b^	2.53 ± 0.04^b^	0.55 ± 0.04^b^	1.79 ± 0.05^b^	1.37 ± 0.06^b^
NL	0.61 ± 0.04	2.41 ± 0.17^b^	0.54 ± 0.05^b^	1.92 ± 0.07^a^	1.32 ± 0.05^b^
NH	0.59 ± 0.05	2.33 ± 0.10^b^	0.51 ± 0.04^b^	1.91 ± 0.09^a^	1.27 ± 0.07^b^
TL	0.53 ± 0.05^c^	2.34 ± 0.10^b^	0.46 ± 0.02^b^	1.99 ± 0.07	1.22 ± 0.07^b^
TH	0.49 ± 0.03^d^	2.19 ± 0.07^b^,^c^	0.43 ± 0.02^a^,^c^	2.14 ± 0.07^c^	1.09 ± 0.05^a^,^d^
ST	0.45 ± 0.04^d^	1.77 ± 0.10^d^	0.41 ± 0.02^c^	1.95 ± 0.13	0.90 ± 0.02^d^

TG, triglyceride; TC, total cholesterol; LDL-C, low-density lipoprotein cholesterol; HDL-C, high-density lipoprotein cholesterol.

a *p* < 0.05.

b *p* < 0.01 compared with NC.

c *p* < 0.05.

d *p* < 0.01 compared with HC.

### MDA and antioxidant enzyme activities in hyperlipidaemic rats

Compared to the NC group, a significant increase in serum MDA concentration and decrease in GSH-Px, SOD and CAT activities were observed in the HC group (Table 5, *p* < 0.01). However, the changes of serum MDA level and SOD and CAT activities were clearly improved by treatment with high-dose of TAE (*p* < 0.05), not by the NeE treatment.

**Table 5.** Effects of different extracts on serum MDA level, SOD, GSH-Px and CAT activities in hyperlipidaemic rats (*n* = 8, mean ± S.E.).

Group	MDA (nmol/mL)	GSH-Px (μmol/mL)	SOD (U/mL)	CAT (U/mg of protein)
NC	2.55 ± 0.09	732.62 ± 37.36	223.46 ± 15.43	17.77 ± 1.00
HC	5.22 ± 0.27^b^	516.98 ± 32.91^b^	172.47 ± 9.2^b^	13.24 ± 0.47^b^
NL	5.00 ± 0.26^b^	537.30 ± 42.21^b^	178.62 ± 10.18	14.13 ± 0.58^b^
NH	5.15 ± 0.26^b^	558.26 ± 41.37^b^	183.81 ± 11.21	14.29 ± 0.45^b^
TL	4.85 ± 0.34^b^	571.16 ± 39.9^b^	186.88 ± 12.22	14.49 ± 0.55^b^
TH	4.24 ± 0.16^b^,^c^	615.69 ± 33.7	206.92 ± 15.38^c^	16.79 ± 0.83^d^
ST	4.10 ± 0.22^b^,^d^	636.44 ± 22.14^c^	205.74 ± 13.78^c^	17.55 ± 0.74^d^

MDA, malondialdehyde; GSH-Px, glutathione peroxidase; SOD, superoxide dismutase; CAT, catalase.

a *p* < 0.05.

b *p* < 0.01 compared with NC.

c *p* < 0.05.

d *p* < 0.01 compared with HC.

### Histopathological analysis

**Figure 4.**
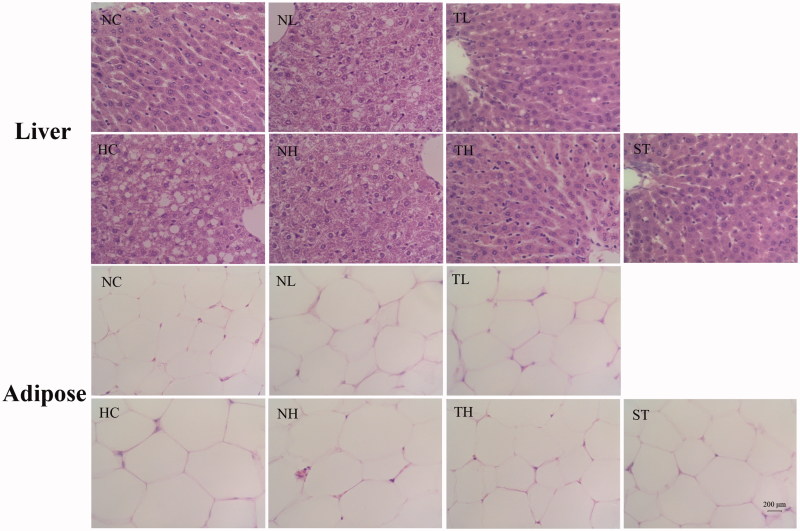
Effects of different extracts on histopathologic changes in the liver and adipose tissue of hyperlipidaemic rats. Original magnification 400 × and HE staining.

Hepatohistological analysis indicated the presence of steatosis and cytoplasmic vacuoles in the livers of hyperlipidaemic rats (Figure 4). Nevertheless, treatment with TAE alleviated the liver lesions in rats. When compared to the NC group, an enlargement of the adipocytes in the adipose tissues from the HC group was observed by light microscopy, while the rats in the TAE and ST treatment groups exhibited a reduction in size of the adipocytes (Figure 4).

### Serum apoB48 in hyperlipidaemic rats

**Figure 5.**
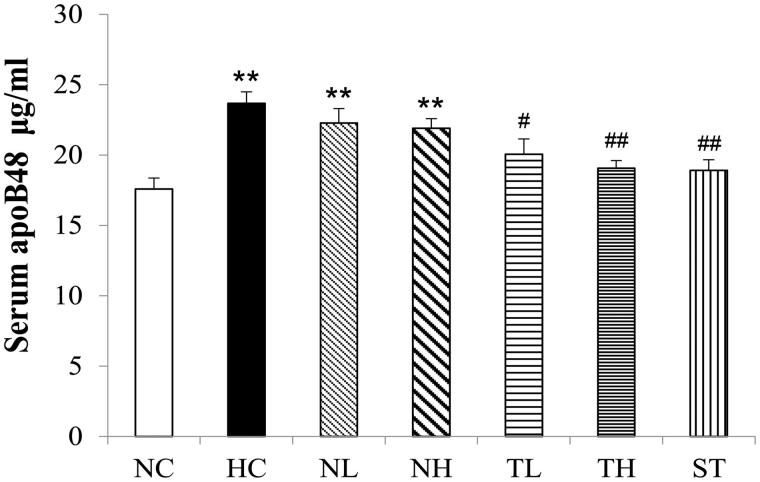
Effects of different extracts on serum apoB48 protein levels in hyperlipidaemic rats (μg/mL, *n* = 8, mean ± S.E.). *p < 0.05, **p < 0.01 compared with NC; #*p* < 0.05, *##p* < 0.01 compared with HC.

Data from Figure 5 indicated that HFD feeding led to an elevation of serum apoB48 level compared to the normal control (*p* < 0.01). However, TAE treatment (200 and 400 mg/kg) decreased the serum apoB48 levels by 15.3 and 19.4%, respectively. High-dose treatment of TAE (400 mg/kg) brought the concentrations back to a level comparable the NC group (*p* < 0.01). No significant effects were observed in rats treated with NeE.

### Intestinal apoB48, MTP protein and mRNA levels in hyperlipidaemic rats

**Figure 6.**
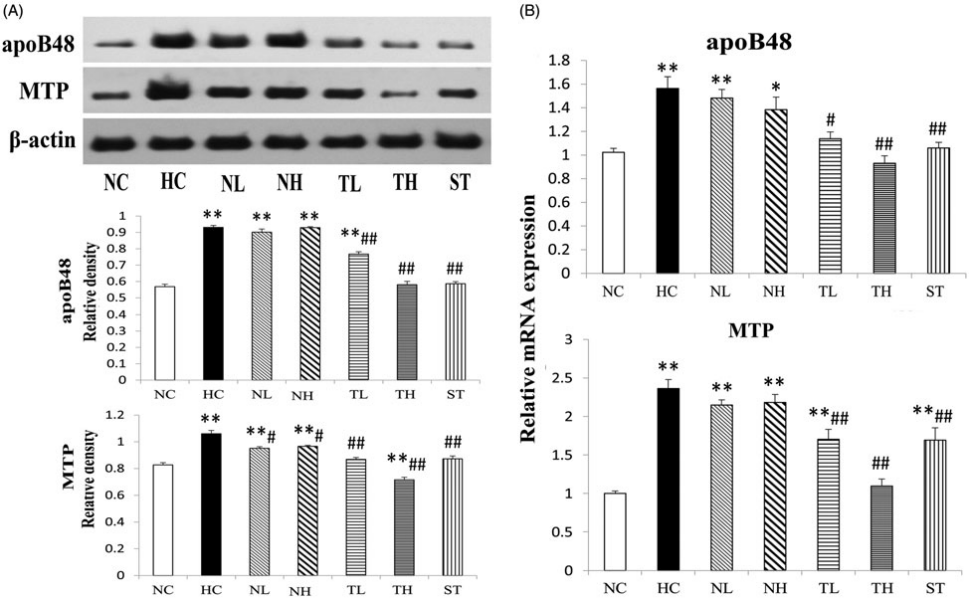
Effects of different extracts on intestinal protein (A) and mRNA (B) levels of apoB48 and MTP protein. Results are expressed as mean ± S.E. of three independent experiments. *p < 0.05, **p < 0.01 compared with NC; #*p* < 0.05, ##*p* < 0.01 compared with HC.

Intestinal apoB48, MTP protein and mRNA levels were presented in Figure 6. HFD feeding resulted in the remarkable elevation of intestinal apoB48 protein and mRNA level to 1.68-fold and 1.56-fold that of the NC group, respectively (*p* < 0.01). Both TAE and simvastatin treatment reduced the increased protein and mRNA level of apoB48 (*p* < 0.05), but NeE group did not show similar effect.

The protein and mRNA expression levels of MTP in HFD-fed rats were dramatically up-regulated by 1.28-fold and 2.36-fold compared to the NC group (*p* < 0.01), while down-regulated levels were obtained in rats with TAE (21.7% and 48.4% decrease at 200 and 400 mg/kg, respectively) and ST administration (*p* < 0.01). NeE treatment led to a significant regulation on MTP protein (*p* < 0.05) and a weak effect on the RNA expression level (not significantly).

### TNF-α and MAPK expressions in hyperlipidaemic rats

**Figure 7.**
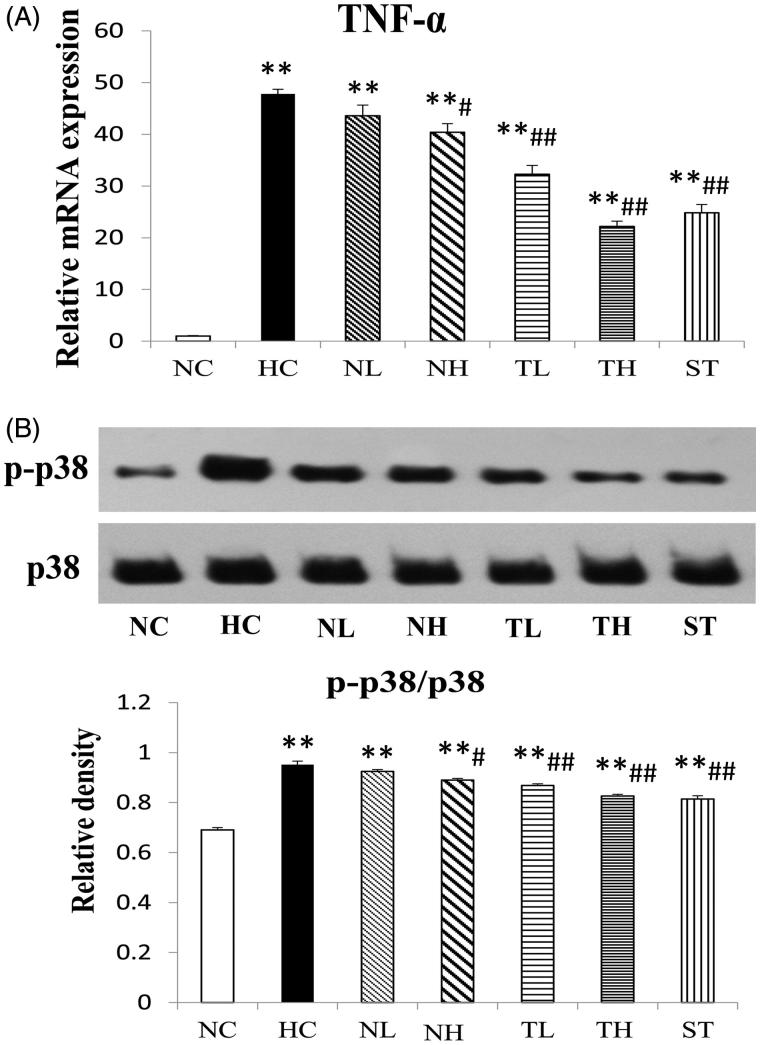
Effects of different extracts on p38 MAPK phosphorylation (A) and TNF-α mRNA levels (B) in hyperlipidaemic rats. P-p38 was determined by western blot. Results are expressed as mean ± S.E. of three independent experiments. *p < 0.05, **p < 0.01 compared with NC; #*p* < 0.05, ##*p* < 0.01 compared with HC.

To demonstrate the effect of TAE on intestinal TNF-α/p38 MAPK pathway, the mRNA of TNF-α and the phosphorylation level of p38 MAPK in intestinal tissue were determined by RT-PCR and western blot experiment, respectively (Figure 7). As expected, the mRNA level of TNF-α in the HC group was up-regulated about 47-fold compared to NC group, while significantly down-regulated by TAE treatment by 36.2 and 56.2% (*p* < 0.01), respectively (Figure 7(A)). The phosphorylation level of p38 MARK were markedly increased about 1.38-fold in the HC group (Figure 7(B), *p* < 0.01), but subsequently decreased after treatment with the plant extracts (*p* < 0.05), especially TAE (TL and TH, 8.8 and 13.2% decrease, respectively, *p* < 0.01). These data indicated *C. paliurus* down-regulated the TNF-α/MAPK pathway in the hyperlipidaemic rodents, which was consistent with the previous study (Ma et al. 2015).

## Discussion

Evidence was available to demonstrate the beneficial effects of *C. paliurus* extracts on hyperlipidaemia (Ren 1994; Chen et al. 2002; Kurihara et al. 2003). However, the active compounds remain unclear. In the present study, a triterpenic acid-enriched fraction of *C. paliurus* leaves exerted the antihyperlipidaemic effect on a HFD-induced hyperlipidaemia rats. The results indicated that TAE could decrease the elevated level of TG, TC and LDL-C, as well as increase the HDL-C level. TAE also ameliorated dyslipidaemia by inhibiting the overproduction of intestinal apoB48. The underlying mechanism might be attributed to downregulating MTP expression level and the TNF-α/p38MAPK signalling pathway.

Dyslipidaemia is mainly resulting from a sedentary life style and fat-rich diets (Naser et al. 2008). Hyperlipidaemia is known to elevate levels of lipid peroxidation, and the products such as MDA has been associated with the deposition of LDL on vascular wall (Delgado Roche et al. 2012). High TC/HDL ratio, the atherogenic index (AI), indicates a higher risk of atherosclerosis development (Xia et al. 2011). Therefore, our study applied HFD feeding to induce dyslipidaemia in rats. TAE exerted the beneficial effects to mitigate dislipidaemia and the antioxidant properties in the HFD-induced hyperlipidaemia rats. These findings further support the antihyperlipidaemic activities of TAE from *C. paliurus* and its potential effect in the treatment of cardiovascular disorder.

Apolipoproteins are the primary organizing protein components of the lipoproteins. ApoB48, in response to dietary lipids, is mainly synthesized in enterocytes and involved in chylomicron assembly (Pal et al. 2005). ApoB48 also has been used as a measure of intestinal TRL particles (Zilversmit 1995). A high concentration of plasma apoB48 is associated with plaques formation that causes vascular diseases and is believed to be a reliable indicator of cardiovascular diseases (Mori et al. 2013). Lipid-lowering agents including simvastatin and natural products like green tea polyphenols have been reported to reduce the level of plasma intestinal-derived apoB48 (Borthwick et al. 2014). Our previous work also revealed *C. paliurus* leaves extract remarkably decreased plasma total- and TRL-apoB48 levels (Ma et al. 2015). In the present study, TAE also improved the elevation of serum apoB48 level, as well as down-regulated the expression level of intestinal apoB48. The antihyperlipidaemic efficacy might be attributed to the TAE inhibitory effect on the intestinal apoB48 overproduction and secretion.

*De novo* synthesized apoB48 molecules are either secreted within chylomicrons or intracellularly degraded by proteases (Chen et al. 1987; Welty et al. 1999). MTP facilitates the translocation of newly synthesized apoB48 to the endoplasmic reticulum, thereby protects apoB from intracellular degradation in the intestine (Hussain et al. 2003). In our study, the decreased expression of MTP might be owing to the down-regulatory effect of TAE on intestinal apoB48 secretion.

Inflammation is an important aetiology underlying lipid metabolism disorders (Chen et al. 2009). TNF-a, an inflammatory cytokine, is one of the agonists of the MAPK pathway and plays a potential role in the metabolism of apoB48 (Yun et al. 2009; Qin et al. 2007, 2010). It is demonstrated that TNF-α stimulation on intestinal apoB48 production and MTP expression might be mediated via the p38 MAPK pathway (Qin et al. 2007). Moreover, MAPKs was reported to interfere with apoB48 production and MTP activity by regulating the expressions of CD36 (Yun et al. 2009), a transmembrane protein involved in lipid absorption (Tran et al. 2011). In the present study, TAE inhibited TNF-α expression and p38 phosphorylation in hyperlipidaemia rats, which was consistent with the previous report (Ma et al. 2015). We speculated that TAE inhibitory effect on intestinal apoB48 overproduction might be partially mediated by the TNF-α/p38 MAPK signalling pathway. Further investigation is warranted to delineate the underlying mechanism(s) associated with the regulation effect of CD36 on apoB48 metabolism.

The main components of TAE were identified according to the retention times and quasi-molecular ion peaks compared with authentic reference compounds. The results revealed the presence of dammarane-, ursane- and oleanane-type triterpenic acids, including arjunolic acid, 2α, 3α, 23-trihydroxyursa-12, 20(30)-dien-28-oic acid, cyclocaric acid B, pterocaryoside B, 2α, 3α, 23-trihydroxyurs-12-en-28-oic acid, 3β, 23-dihydroxy-12-ene-28-ursolic acid, hederagenin and oleanolic acid, in the TAE fraction. Arjunolic acid and 3β, 23-dihydroxy-12-ene-28-ursolic acid possess an inhibitory activity of cholesterol acyltransferase, and exerted antihyperlipdemic effect (Kim et al. 2005; Khaliq et al. 2013; Han et al. 2014). In addition, oleanolic acid has been reported to decrease serum lipids and reduce hepatic lipid accumulation (Wang et al. 2013b). Moreover, 3β, 19*α*-dihydroxyurs-12, 20(21)-diene-28-oic acid can reduce the elevated lipid level in streptozotocin-induced diabetic mice (Gutiérrez et al. 2009). Echinocystic acid showed inhibitory effect on cholesterol acyltransferase activity (Han et al. 2014). Our results further support that triterpenic acids can ameliorate hyperlipidaemia. Taken together, it is reasonable to speculate that the triterpenic acids might be responsible for the lipid-lowering effects of *C. paliurus* leaves. Further studies are necessary to investigate the structure-activity relationships and the antihyperlipidaemic mechanism(s).

## Conclusion

The triterpenic acid-enriched fraction of *C. paliurus* leaves could mitigate dyslipidaemia by decreasing the atherogenic lipid levels in serum and liver, and increasing the antioxidant enzyme activities. The underlying antihyperlipidaemic effect may be partially mediated by inhibiting the production of intestinal apoB48 through the TNF-α/p38 MAPK signalling pathway.

## Supplementary Material

Zhiqi_Yin_et_al_Supplemental_content.zip
