# Supplementary material for: Antihyperlipidaemic effect of triterpenic acid-enriched fraction from Cyclocarya paliurus leaves in hyperlipidaemic rats
Source: Pharm Biol. 2017 Jan 31;55(1):712–21. doi: 10.1080/13880209.2016.1267231 (PMC6130609; doi:10.1080/13880209.2016.1267231)

Supplementary material

Fig. S1 Respective HPLC-MS spectra of each compound. The quasi-molecular ion peak ($[M-H]^-$) was marked with an arrow.

Arjunolic acid

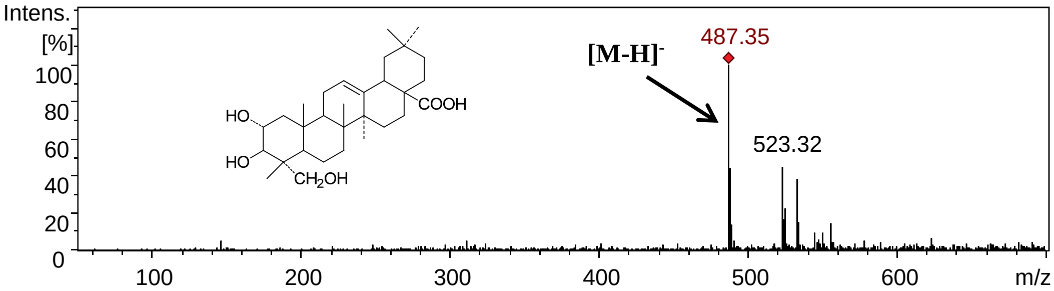

2α, 3α, 23-trihydroxyursa-12, 20(30)-dien-28-oic acid

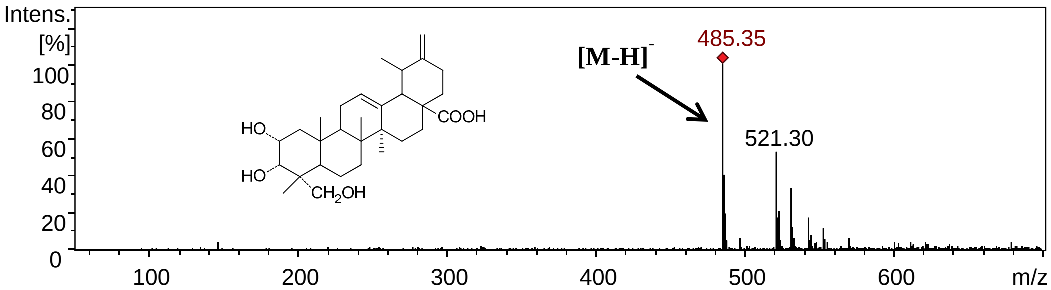

Cyclocaric acid B

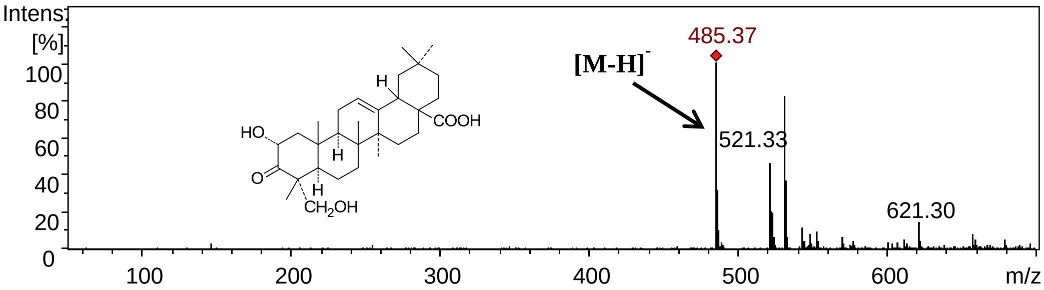

Pterocaryoside B

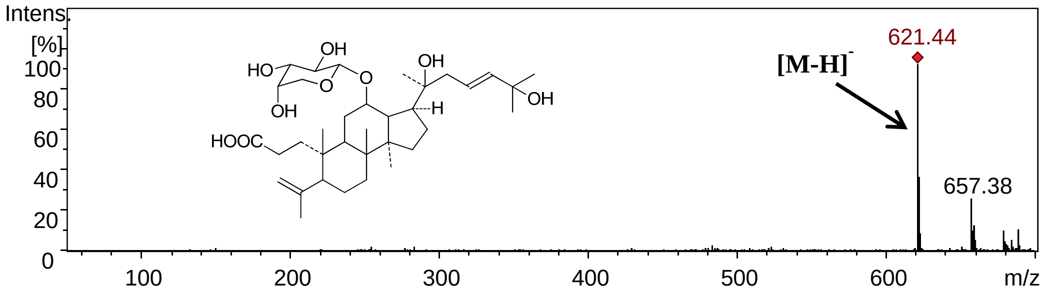

2α, 3α, 23-trihydroxyurs-12-en-28-oic acid

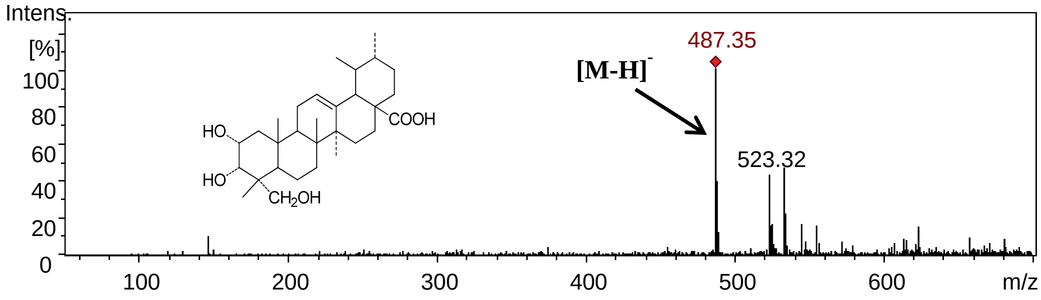

3β, 23-dihyroxy-12-ene-28-ursolic acid

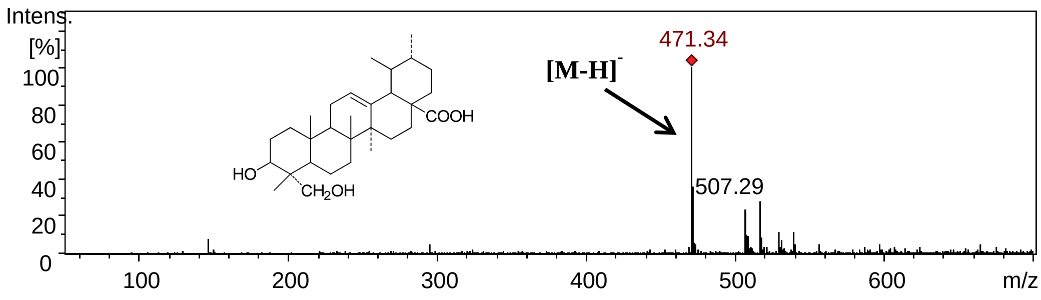

Hederagenin

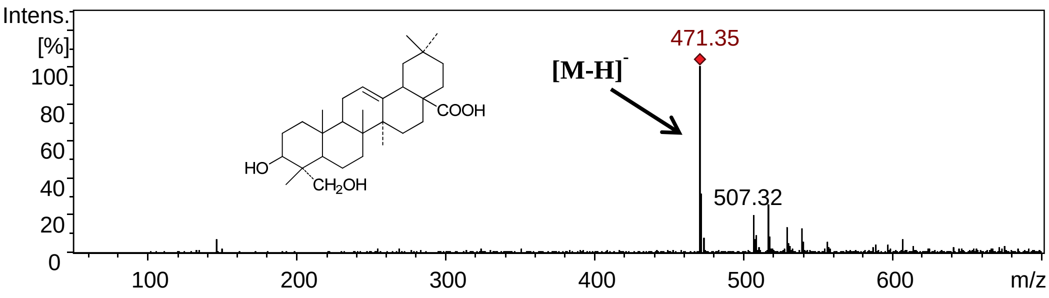

Oleanolic acid

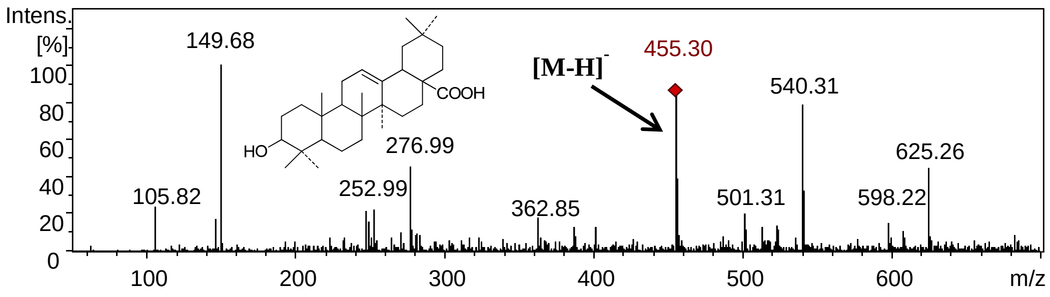

Supplement: Zhiqi_Yin_et_al_Supplemental_content.zip [file IPHB_A_1267231_SM8651.zip › Zhiqi Yin et al Supplemental content.pdf]
